# A latent class analysis of community-based rehabilitation needs among Chinese older adults: a mixed study protocol

**DOI:** 10.3389/fpubh.2023.1301752

**Published:** 2024-01-12

**Authors:** Lei Xu, Caixiu Xue, Ke Yang, Lingyun Chen, Xidong Chen, Xiaohui Xie, Jia Wang, Xueting Wang, Lianhong Wang

**Affiliations:** ^1^Nursing Department, Affiliated Hospital of Zunyi Medical University, Zunyi, Guizhou, China; ^2^Nursing College, Zunyi Medical University, Zunyi, Guizhou, China; ^3^Department of Neurological Rehabilitation, Geriatric Rehabilitation Hospital Affiliated Hospital of Zunyi Medical University, Zunyi, Guizhou, China

**Keywords:** community-based rehabilitation, rehabilitation needs, latent class analysis, health promotion, public health informatics

## Abstract

**Background:**

Geriatric diseases (e.g., chronic diseases and geriatric syndromes) may result in impaired physical performance and a decline in the quality of life. The results of previous studies reported the positive effects of comprehensive community-based rehabilitation (CBR) services on physical and social functioning and psychosocial wellbeing. However, to provide adequate and personalised rehabilitation services, it is essential to understand the needs of the older adults population. There have been no studies on the need for CBR in older adults populations that consider their heterogeneity. Therefore, high-quality studies are required to recognise the heterogeneity and latent classes of CBR needs in older adults population groups. This study aims to identify the heterogeneity of the rehabilitation needs of older adults in the community and explore whether older adults with similar characteristics have similar needs through a cross-sectional survey and latent class analysis (LCA) to provide support for personalised rehabilitation services.

**Methods:**

The study is structured into four phases. The first phase will focus on constructing a comprehensive questionnaire to assess rehabilitation needs. In the second phase, a pilot study will be conducted to evaluate the reliability and validity of the completed questionnaire. This step ensures the robustness of the instrument for data collection. The third phase will involve cross-sectional surveys using the finalised questionnaires to collect the necessary data from the targeted population. The fourth phase will focus on conducting LCA to determine the CBR needs of the older adult population.

**Discussion:**

The results of this study will provide novel and critical information for a better understanding of the rehabilitation needs, potential categories, and influencing factors of older adults in the community. The study will be conducted in Guizhou Province in western China, where economic and social development is relatively low, and the results will inform and benefit other regions and developing countries facing similar challenges. However, because of the complete social security and rehabilitation service systems in developed areas, our research results may not fully reflect the situation in these areas. Future studies may need to be conducted in places with different levels of social development.

**Clinical trial registration:**

https://www.chictr.org.cn/showproj.html?proj=191398, ChiCTR2300071478.

## 1 Introduction

### 1.1 Background

Population ageing is accelerating worldwide, and this demographic transition presents a formidable challenge in managing global public health policies. In China, the estimated older population is expected to increase from 267 million in 2021 to 400 million in 2035, constituting 30% of the total population (1). As people age, their functional performance and intrinsic abilities decline (2), leading to an increased occurrence of chronic illnesses, geriatric syndromes, and multiple medical and psychosocial conditions (3). Studies in China have indicated that the prevalence of chronic diseases in older adults is 69.13% (4), with multiple chronic diseases, pain, mobility disorders, and depressive disorders occurring in 45.5% (5), 30% (6), 19.6% (7), and 36.8% (8) of older adults, respectively, which can profoundly influence their health status and quality of life (9). These findings indicate the importance of offering rehabilitation services to older adults to support their physical, psychological, and social wellbeing.

Among the many forms of rehabilitation service delivery, community-based rehabilitation (CBR) is an effective strategy that addresses disability, rehabilitation, equalisation of opportunities, poverty reduction, and social inclusion of older adults (10). This approach not only offers convenience and feasibility but also preserves older people’s existing social networks, diminishes medical costs, and is widely embraced by this demographic (11, 12). In this process, the success of CBR can be gauged by how well the community can provide customised rehabilitation services for senior citizens (13–15). To achieve this, it is crucial to correctly understand their rehabilitation needs. However, current studies focused on understanding the rehabilitation of individual diseases or repeatedly investigating the overall rehabilitation needs of the entire older adults population and, based on this limited information, used the same method to guide CBR in the older adults population (16–18). Meanwhile, because rehabilitation needs tend to co-occur and heterogeneity exists within a population, a single-need assessment of rehabilitation services is unsuitable for older adults with multiple chronic diseases or various physiological, psychological, and social conditions. Moreover, owing to individual differences and heterogeneity, the scientificity and rationality of providing the same CBR services to older adults need to be verified. Hence, the description of the complete picture of the CBR needs of the older adults population needs to be explored to provide personalised CBR services based on different CBR need subtypes in older adults individuals or groups.

Latent class analysis (LCA) is an effective tool for understanding the overall needs for CBR in older adults and identifying the different CBR need subtypes. LCA describes population heterogeneity in terms of differences across individuals in a set of behaviours or characteristic statistical methods, which is called the person-centred approach (19). Owing to these advantages, it has been used in many fields, such as medicine, sociology, and psychology (20–22), to identify unobserved subgroups, especially potential needs types (23–25). Using LCA, researchers may be able to categorise the CBR needs of older adults into different latent categories and make each category representative of the rehabilitation needs of people with similar characteristics. At the same time, if researchers add covariates, they can also obtain a clearer picture of the factors that influence the CBR needs of older adults. To the best of our knowledge, no LCA studies have been conducted to determine the needs of older adults with CBR.

This study aims to fill existing gaps by conducting a cross-sectional study of the CBR needs of older adults in China and using LCA to elucidate the CBR needs of older adults, including the different need subtypes and influencing factors for this population. The results of this study will positively impact the formulation of targeted CBR policies for older adults and the provision of personalised CBR services.

### 1.2 Theoretical framework

**Figure 1:**
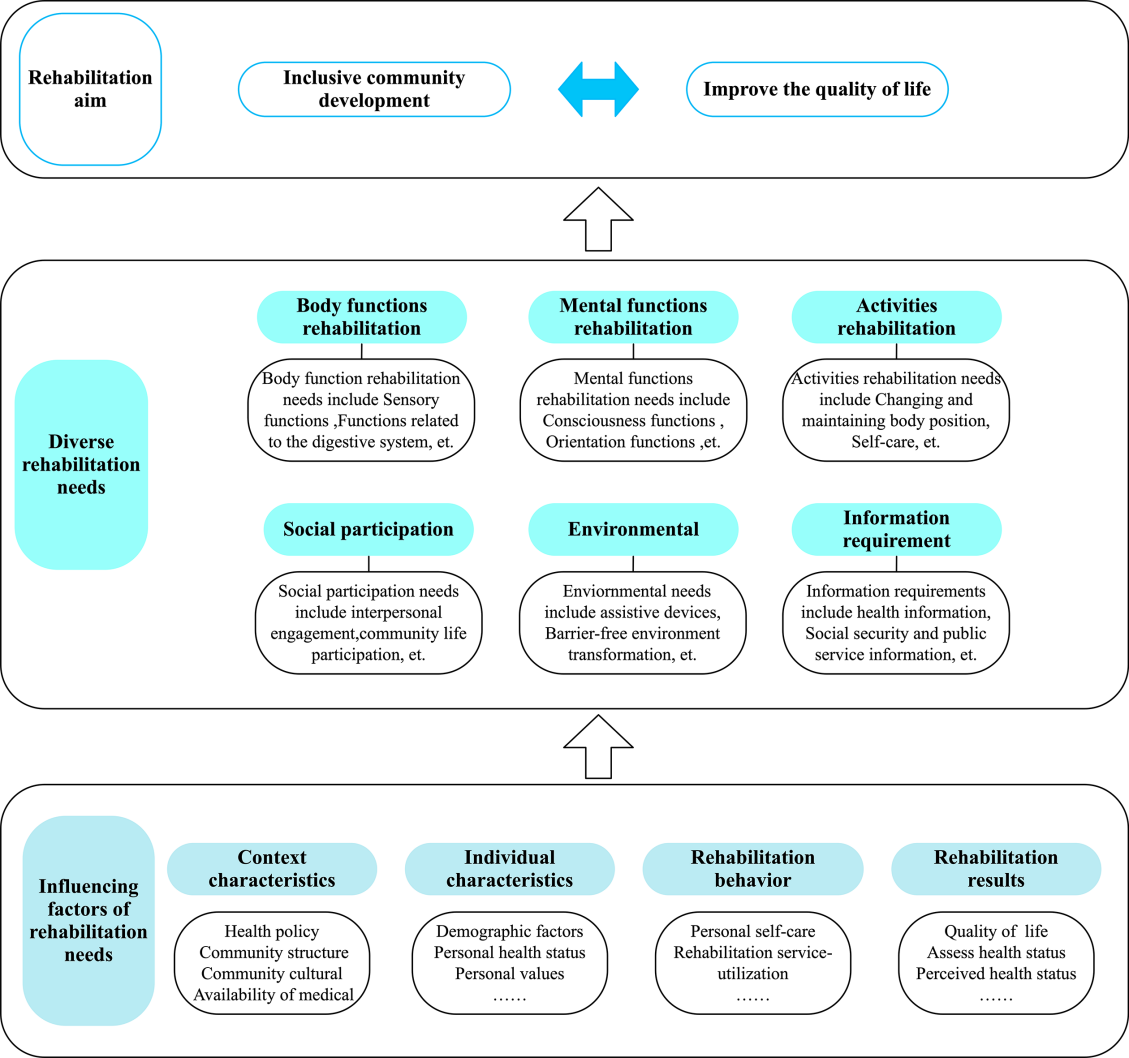
Theoretical framework of multiple needs for community-based rehabilitation in older adults based on the latent class model.

Generation of the CBR needs for older adults stems from their functional performance and intrinsic abilities. To enhance our understanding of the CBR needs of older adults, we have integrated three theoretical frameworks: International Classification of Functioning, Disability, and Health (ICF) (26), Guidelines for Community-Based Rehabilitation, and Behavioural Model of Health Services Use (BMHSU) to guide our study (Figure 1). Based on the ICF, we can analyse the physical, psychological, social, and environmental rehabilitation needs of older adults. By establishing a needs assessment mechanism based on the ICF, we laid the foundation for developing a comprehensive CBR implementation plan (27). Simultaneously, to better understand the rehabilitation needs of the older population at the community level, we implemented the Guidelines for Community-Based Rehabilitation. Combining this guide with the ICF can help us understand and explore the CBR needs of older populations from various perspectives, which results in the most critical component of our theoretical framework (Figure 1): diverse rehabilitation needs.

To better understand the factors affecting the CBR needs of older adults, we introduced BMHSU, a classical theoretical model in the field of public health. This model explores the relationship between individual factors and health services (28), elucidating intricate interactions among contextual characteristics, individual characteristics, health behaviours, and health outcomes. Successfully applied in studying older adults’ inclinations toward institutional care, utilisation of long-term care services, community health management, and utilisation behaviour, BMHSU provides valuable insights into individuals’ demand for health services (29–31). Based on the four dimensions offered by BMHSU, we will explore the factors that affect the CBR needs of older adults through a practical setting of influencing factors. Integrating these three theories enables a comprehensive understanding of the CBR needs of older adults, a perspective considering diverse needs categories and influencing factors.

### 1.3 Study questions

This study hypothesises that older adults have physiological, psychological, and social participation and environmental and information needs in CBR. Since older adults have multiple needs at the same time, there may be several fixed combinations of these needs in the older adults population (e.g., “Physiological-psychological” combination, “socially-participation-environment” combination). Each older adults person will fall into one of these categories, and contextual characteristics, individual characteristics, health behaviours, and health outcomes influence these combinations.

We aim to address the following three research questions: (i) When older adults individuals have multiple rehabilitation needs, is it more likely that these needs will be combined in a specific manner rather than randomly? (ii) Do older adults with similar characteristics have similar needs? (iii) What key factors are associated with the needs of older adults with multiple CBR needs?

## 2 Methods

**Figure 2:**
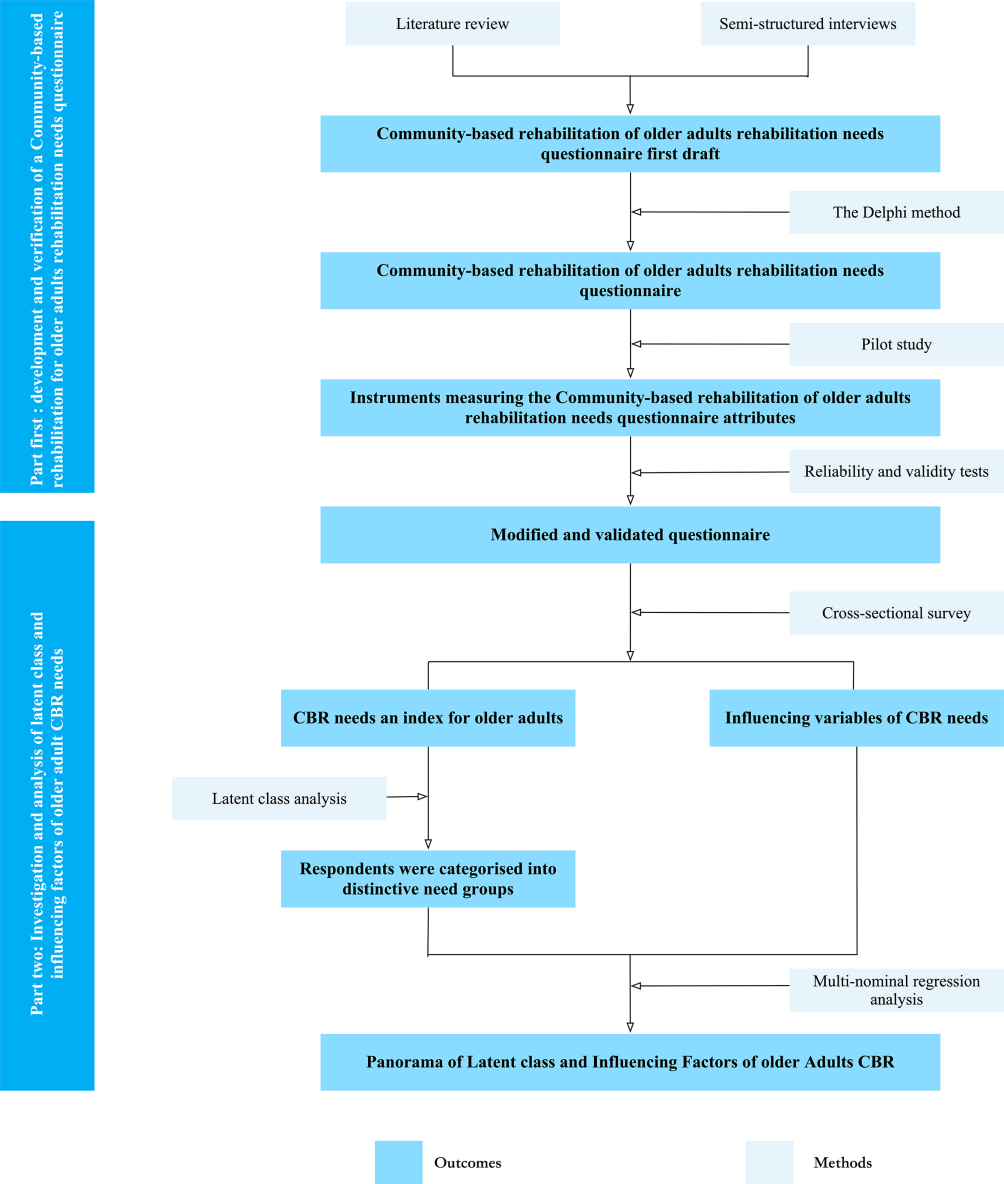
The flowchart describes the main results and critical approaches of the two parts of the current study.

This study will be a cross-sectional survey guided by our incorporated theoretical framework. This sequential exploratory mixed-methods study is categorised into four phases (Figure 2), which are as follows:

The first part is the development of CBR for older adults’ rehabilitation needs questionnaire, and the first phase will be the development of older adults’ CBR needs questionnaire based on our theoretical framework. This phase includes a literature review, semi-structured interviews, and the Delphi method. The second phase will be a pilot study to verify the older adults’ CBR needs questionnaire and will include validation of the other measurement scales used in this study. This order is commonly employed for questionnaire preparation and verification (32–34).

The second part will involve collecting sufficient data to conduct LCA through a cross-sectional study design. The third phase is the main study, mainly a cross-sectional survey based on the questionnaire used in the pilot study to help us obtain the data needed for LCA. The fourth phase will be LCA, which aims to identify the diversity of older adults’ CBR needs, uncover latent relationships, and determine the factors influencing their needs.

### 2.1 Phase 1 development of older adults’ CBR needs questionnaire

#### 2.1.1 Literature review

First, to determine the possible dimensions and items in the scale, we will review the literature extensively to better understand the relevant literature and study status affecting the rehabilitation needs of older adults in community-based settings. Literature on the rehabilitation needs of community-dwelling older adults will be searched through PubMed, Embase, Web of Science, CENTRAL, CINAHL, Physiotherapy Evidence Database, CNKI, and other databases. The key search terms will include a combination of “Aged/Elderly/Older adults/Older persons/Aged people/Older adult,” “Community rehabilitation/CBR/Community-based rehabilitation/Home rehabilitation,” and “Need/Demand/Requirement.” The studies included should focus only on the CBR needs. Duplicate publications, studies that did not separate the range of the qualitative data, and non-English or Chinese studies will be excluded. Based on a comprehensive review of the existing literature combined with our theoretical framework, we will construct a questionnaire on body function rehabilitation, mental function rehabilitation, individual activities, social participation, and environmental rehabilitation. Researchers have developed an information needs module to understand the needs of older adults and help them integrate into the community. This module combines content from the Guidelines for Community-Based Rehabilitation (10) and the ICF environment module to reduce the impact of the digital gap. The goal is to implement rehabilitation services through inclusive community development. The primary item pool of the needs questionnaire is shown in Table 1.

**Table 1:** The needs questionnaire preparation indicators.

Primary index	Secondary index	Category description (ICF code and name)
Body function	Vision and related functions	b210 Visual functions b215 Functions of structures adjoining the eye b220 Sensations associated with the eye and adjoining structures
	Hearing and vestibular functions	b230 Hearing functions b235 Vestibular functions b240 Sensations associated with hearing and vestibular function
	Pain	b280 Sensation of pain
	Voice and speech functions	b310 Voice functions b320 Articulation functions b330 Fluency and rhythm of speech functions b340 Alternative vocalisation functions
	Heart functions	b410 Heart functions
	Functions of the respiratory system	b440 Respiration functions b445 Respiratory muscle functions
	Swallowing function	b510 Ingestion functions
	Defecation functions	b525 Defecation functions b620 Urination functions
	Weight maintenance functions	b530 Weight maintenance functions
	Functions of the joints and bones	b710 Mobility of joint functions b715 Stability of joint functions b720 Mobility of bone functions
	Muscle functions	b730 Muscle power functions b735 Muscle tone functions b740 Muscle endurance functions
	Movement functions	b750 Motor reflex functions b755 Involuntary movement reaction functions b760 Control of voluntary movement functions b765 Involuntary movement functions b770 Gait pattern functions
Mental functions	Cognitive function	b110 Consciousness functions b114 Orientation functions b140 Attention functions b144 Memory functions b164 Higher-level cognitive functions
	Sleep functions	b134 Sleep functions
	Psychomotor functions	b147 Psychomotor functions
	Emotional functions	b152 Emotional functions
Individual activities	Changing and maintaining body position	a410 Changing basic body position a415 Maintaining a body position a420 Transferring oneself
	Carrying, moving, and handling objects	a430 Lifting and carrying objects a435 Moving objects with lower extremities a440 Fine hand use a445 Hand and arm use
	Walking and moving	a450 Walking a455 Moving around a460 Moving around in different locations
	Self-care	a510 Washing oneself a520 Caring for body parts a530 Toileting a540 Dressing a550 Eating a560 Drinking a570 Looking after one’s health
Social participation	Interpersonal interactions	p710 Basic interpersonal interactions p720 Complex interpersonal interactions
	Interpersonal relationships	p730 Relating with strangers p740 Formal relationships p750 Informal social relationships p760 Family relationships p770 Intimate relationships
	Community life participation	p910 Community life
	Recreation and leisure	p920 Recreation and leisure
Environment	Use of assistive devices	e115 Products and technology for personal use in daily living e120 Products and technology for personal indoor and outdoor mobility and transportation e125 Products and technology for communication
	Cultural and entertainment	e140 Products and technology for culture, recreation, and sport
	Barrier-free environment transformation	e150 Design, construction and building products, and technology of buildings for public use e155 Design, construction and building products, and technology of buildings for private use
Information	Social security and public service information	e545 Civil protection services, systems, and policies e575 General social support services, systems, and policies
	Health-related information	e580 Health services, systems, and policies
	education and training information	e585 Education and training services, systems, and policies
	Laws and policies protect relevant information	e550 Legal services, systems, and policies e595 Political services, systems, and policies
	Social engagement or empowering information	e555 Associations and organisational services, systems, and policies e560 Media services, systems, and policies

#### 2.1.2 Semi-structured interviews

Semi-structured face-to-face interviews will be conducted with older adults individuals with CBR needs to ensure the alignment of items with their respective dimensions and generate valid measurements (35). Participants will be selected through purposeful sampling, adhering to specific criteria for inclusion in the interviews: (1) age ≥ 60 years, (2) either currently undergoing or on the verge of receiving such services, (3) the ability to self-report, characterised by sufficient language understanding and expression skills, and (4) received informed consent from both participants and their families to willingly participate in the study. Sample size will be determined following the principle of data saturation, with an estimated requirement of approximately 20–25 respondents to achieve information saturation. In instances where saturation is uncertain, supplementary interviews will be conducted (36). Two trained individuals will interview each participant at their chosen location. Written informed consent will be obtained before each interview, and the interview sessions will be recorded for an average duration of approximately 30 min.

Participants will be queried on various aspects, including (1) How is your health now? (2) What inconveniences will you encounter during CBR? (3) What are your needs when receiving CBR services? (4) What challenges do you expect when receiving community CBR services? (5) What exceptional help do you require? and (6) What are your suggestions for improving CBR services?

The interview recordings will be transcribed into Word documents for subsequent analyses. Employing the Colaizzi content analysis method, the two researchers will analyse, summarise, encode, compare, confirm, and refine the emergent themes. The questionnaire items will be supplemented based on the results of the qualitative research combined with the primary entry pool.

We will filter our pool of entries based on a literature review and semi-structured interviews to form the first draft of our questionnaire, which will be screened and supplemented by a Delphi study.

#### 2.1.3 The Delphi method

The Delphi method is intended to score and guide the importance and feasibility of constructing an older adult’s CBRN-Q (37).

The researchers will first establish an expert group that includes rehabilitation medicine doctors, geriatric medicine experts, geriatric nurses, and rehabilitation nursing experts. Members of this team must have >10 years of clinical experience and intermediate or higher professional titles. More than 15 experts will be involved in this stage (38).

The survey will include an expert letter questionnaire conducted via e-mail correspondence, a questionnaire that includes the study background, an expert general situation questionnaire, and the main body of the questionnaire. The expert will evaluate the importance (rated on a Likert scale from very important, 5, to unimportant, 1) and feasibility (very low to too much) of each item in the CBR needs questionnaire for older adults. The researchers will summarise, screen, and modify the opinions put forward by experts on older adults’ CBR needs questionnaire items, request the experts for consultation again, and repeat the process until the experts stop contributing their opinions (39). Entries that meet the following criteria will be retained: the mean value of the importance assignment X > 3.5 and the coefficient of variation (CV) < 0.25 (40).

Finally, the research group will discuss expert opinions and suggestions to further improve the older adults’ CBR needs questionnaire. Simultaneously, since the core of the study is the model construction and analysis based on LCA, there should be a clear boundary value for both the scale and items used so that the data can be easily converted into binary variables for analysis.

### 2.2 Phase 2 pilot study

#### 2.2.1 Study sample and sample size

Using purposive sampling, we will conduct a questionnaire survey on older adults in Zunyi, Guizhou Province. The sample size for questionnaire verification should be 10 times the number of questionnaire items (41). Because the questionnaire we developed is estimated to have 40–45 items, we will need at least 450 participants. Considering a 10% loss to follow-up rate, we determine that the sample size of the pre-trial is 500 participants.

#### 2.2.2 Participants

The target population includes the general population that meets the following inclusion criteria:

1. Aged ≥ 60 years;
2. Community-dwelling residents for at least 1 year;
3. Ability to communicate with the researchers and express themselves;
4. Provided written informed consent.

The exclusion criteria are as follows:

1. Older adults with cognitive impairment or major mental disorder;
2. Palliative care (life expectancy <6 months);
3. Permanently bedridden.

#### 2.2.3 Data collection

Specially trained researchers will work closely with community workers to collect data from the community. First, the researchers will determine the eligible participants according to the inclusion and exclusion criteria through the community health records. The community staff will then contact eligible participants and agree on the investigation time and location based on their initial consent. The place to conduct an investigation is usually the community office or participants’ homes. Subsequently, the two researchers will meet with the participants at an agreed time and place. The researchers will talk to the older adults or their caregivers to explain the purpose of the study and the concept of CBR. Their power includes privacy protection and the termination of investigations at any time. Individuals willing to participate will provide written informed consent before completing the survey. Finally, one of the researchers will send an e-questionnaire QR code prepared in advance to qualified participants. The questionnaire will be completed after the code is scanned using a smartphone. Our researchers will help older people who have difficulty using smartphones complete and submit the questionnaire. After completing the questionnaire, we will pay the participants a particular reward or an equivalent gift (approximately $4–5). Data will be extracted using a questionnaire platform and sorted into Microsoft Excel. The instruments used are as follows:

##### 2.2.3.1 Sociodemographic characteristics

Demographic characteristics will be measured using a personal information table developed by the researchers, including age, nationality, education, weight, height, marital status, residence, children, income, whether they have chronic diseases, and the type and number of chronic diseases.

##### 2.2.3.2 Older adults’ CBRN-Q

The questionnaire developed by the researcher in the first phase mainly included body function needs, mental function needs, individual activities, social participation, environmental needs, and information needs.

##### 2.2.3.3 Activities of daily living scale

The ADL scale measures the self-care abilities of older adults. It includes the physical self-maintenance (PSMS) and instrumental ADL (IADL) scales, comprising 14 items. The PSMS has six items for ADL—such as going to the toilet, eating, dressing, and grooming—while the IADL has eight items for using tools, such as making phone calls, shopping, doing housework, and taking medicine. Each item is rated on a scale of 1–4, with 1 being completely able to do it yourself and 4 being completely unable to do it yourself. The total score ranges from 14 to 56 points, with 14 indicating the complete self-care ability, 15–21 indicating decreased self-care ability, and ≥ 22 showing worse self-care ability in daily life. Higher scores indicate worse self-care ability. Cronbach’s alpha value is 0.884 (42).

##### 2.2.3.4 Tilburg frailty indicator (TFI)

Compiled by Dutch scholars Gobbens et al. (43), TFI is mainly used to assess the frailty status of older adults. Chinese scholars Xing et al. (44) conducted cultural debugging and translation of the original scale, resulting in a new version of the TFI, including three dimensions of physical frailty (eight items), psychological frailty (four items), and social frailty (three items), with a total of 15 items. The symptoms mentioned in the presence of items are scored 1 point, while those not present are scored 0 points. The total score is 15 points, and TFI ≥5 points are judged as frailty. Cronbach’s alpha value is 0.686 (44).

#### 2.2.4 Data analysis

This phase aims to test the reliability and validity of the older adults’ CBRN-Q in older adults. Moreover, we will retest the reliability of the ADL and FEI in the population we use. Data will be analysed using SPSS (version 29) and R software.

##### 2.2.4.1 Reliability analysis

In the reliability analysis, we will mainly test Cronbach’s alpha values to test the internal consistency of CBRN-Q and the sub-questionnaire we developed. At the same time, we will re-examine the ADL and FEI scales in the older adults population. It is considered acceptable with a threshold of Cronbach’s α ≥ 0.70 (45).

##### 2.2.4.2 Validity analysis

Exploratory factor analysis (EFA) and confirmatory factor analysis (CFA) will be used to assess the construct validity of the CBRN-Q. The collected samples will be randomly categorised into two independent subsets for EFA and CFA.

Before performing the EFA, we will test whether the data can be factor-analysed using Bartlett’s sphericity test and the Kaiser–Meyer–Olkin (KMO) test. The probability of Bartlett’s Test of Sphericity <0.05 or KMO >0.7 indicates that EFA can be performed (46). We will perform EFA using principal component analysis, oblimin rotation, and parallel analysis. We will keep entries with an item factor load >0.4 and no more than one factor (47, 48).

CFA will verify the factor structure of the CBRN-Q based on maximum likelihood estimates. CFA will use three indexes: comparative fit index (CFI), root-mean-square residual (SRMR), and root-mean mean-square error of approximation. CFI value ≥0.90 indicates an acceptable model fit, and ≤ 0.08 indicates a good fit; similarly, SRMR value ≤0.08 indicates a good fit (46).

### 2.3 Phase 3 main study

#### 2.3.1 Study design and setting

This cross-sectional observational study will be conducted in Guizhou Province, China. This study covers six county-level cities (Guiyang, Zunyi, Liupanshui, Anshun, Bijie, and Tongren) and three autonomous prefectures (Qiandongnan, Qiannan, and Qianxinan). This province is located in southwest China, with a population of over 38 million, of which over 60 years old population is more than 5.93 million. Based on the 2022 data, this region faces several significant challenges compared to China’s overall development, including a notable acceleration in population ageing, a modest level of social development (as evidenced by an urbanisation rate of 54.81%, which is below the national average of 65.20%), and a relatively underdeveloped economy (with a *per capita* GDP of US$ 7,180, representing only 61.03% of China’s average *per capita* GDP).

#### 2.3.2 Participants

Consistent with the selection criteria of our pilot study participants.

#### 2.3.3 Sampling

LCA model building requires numerous parameters, resulting in a sample size that depends on the necessary model analysis. Therefore, standard sample size calculations cannot comply with LCA requirements. For LCA, some researchers have suggested a minimum sample size of 500 (49), whereas others recommend a minimum sample size ranging from 300 to 1,000 (50, 51); therefore, we plan to recruit at least 4,000 participants. The sample size will satisfy the analytical requirements.

A probability-proportionate-to-size (PPS) sampling method will be used to select participants from Guizhou Province. PPS sampling requires the number of clusters to be not less than 30. In this study, we will assume the selection of 50 groups, with 4,000 people categorised into 50 groups, each containing 80 participants. These 50 groups will be drawn from six county-level cities and three autonomous prefectures. The number of clusters (*n*) in each of six county-level cities and three autonomous prefectures will be calculated as follows: ① The population aged ≥60 years in Guizhou Province (*N*) divided by 50 and ② The number of clusters (*n*) will be equal to the number of integer multiples of n/50 in the corresponding city cumulative population interval. Then, *n* natural villages/streets in the corresponding prefecture-level cities will be randomly selected as *n* clusters relative to the cities. Detailed information on each prefecture-level city cluster is presented in Table 2.

**Table 2:** Clusters in six county-level cities and three autonomous prefectures.

Township	Population	Accumulative population	*n* of clusters
1.Guiyang	795,475	795,475	7
2.Zunyi	1,126,525	1,922,000	9
3.Liupanshui	409,888	2,331,888	3
4.Anshun	392,951	2,724,839	3
5.Bijie	954,763	3,679,602	8
6.Tongren	579,146	4,258,748	5
7.Qiandongnan	642,509	4,901,257	5
8.Qiannan	585,724	5,486,981	5
9.Qianxinan	443,626	5,930,607	4
Total	*N*	5,930,607	50

#### 2.3.4 Data collection methods

##### 2.3.4.1 Data collection

The data collection method will be consistent with that of our pilot study.

##### 2.3.4.2 Data management

The entire research team will be trained in data collection and storage. As our data will be obtained through electronic surveys, we will ensure the quality of questionnaire submission by limiting the occurrence of missing values and outliers in the electronic questionnaire. After de-tagging, all uploaded data will be stored in a dedicated encrypted database, and only the lead researcher will have access to the complete data. The data analyst only will have access, and the other researchers will not have access to the data unless specifically authorised by the lead researcher. All data management processes will comply with the privacy protection principles and the Data Security Law of the People’s Republic of China.

#### 2.3.5 Quality control

1. Before the formal investigation, we will train the community and street staff to unify the standards and investigation methods to ensure that the investigation data are objective and accurate.
2. To ensure that the survey results are true and accurate for items that older people do not understand during the filling process, we will use a unified language to explain them.
3. The questionnaire star will be set up to ensure that each option is filled in to ensure the completeness of the content and efficiency of the recovery.
4. The obtained data will be double-checked; if there is any difference, the data will be checked with the original data and corrected to ensure accuracy.

#### 2.3.6 Data preprocessing

We will sort the collected questionnaires and upload the data. We will use Excel to establish the database and SPSS (version 29) for preliminary data analysis. Statistical significance is set at *p* < 0.05. We will use descriptive statistics for our general data, such as frequency and ratio. In addition, we will conduct data cleaning, outlier processing, data conversion, and other preprocessing of the data.

### 2.4 Phase 4 LCA

**Figure 3:**
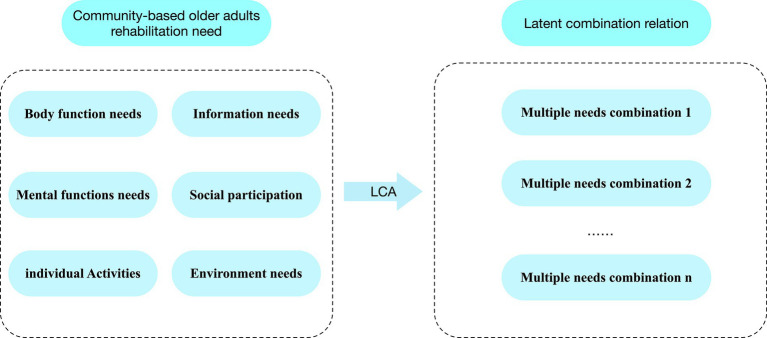
Concept map of a latent class of rehabilitation needs of older adults.

In this phase, we will conduct LCA. By analysing CBRN-Q filled out by the respondents, we will identify exactly which CBR requirement types they have (including body function rehabilitation, mental function rehabilitation, individual activities, social participation, information needs, and environmental rehabilitation), and according to these types of demand at the group level is divided into several different categories, the basic framework is shown in Figure 3 to understand further the diversity of the rehabilitation needs of older adults in the community-based and to classify them accurately. We will use MPlus software (version 7.0) for LCA with the collected data through parameterisation, estimation, evaluation, classification, and interpretation of the results. Simultaneously, we will verify the factors that influence the needs of older adults by adding covariates.

#### 2.4.1 Model parameterisation

LCA contains manifest variables, latent variables, latent-class probabilities, and conditional probabilities. The latent class probabilities represent the probability that an observation object randomly selected from the sample belongs to latent class *t*, and the sum of all latent class probabilities is 1. The conditional probability represents the probability that a sample is randomly selected from the latent class and answered using the manifest variable. The greater the conditional probability value, the greater the influence of the latent variable on the external variable (52). In this step, we will convert the categorical variables processed in the cross-sectional survey into parameters required for model construction, which is the basis of LCA.

#### 2.4.2 Parameter estimation

After parameterisation, we will use the maximum likelihood method for model estimation. Currently, the commonly used methods are expectation maximisation (EM) and the Newton–Raphson method. Since EM is not affected by initial value selection, it is now widely utilised (53). In our study, we will also use this method, starting from an initial model with only one class for all samples and using a step-by-step iterative method to gradually increase the number of classes until the optimal model is found.

#### 2.4.3 Evaluation and classification

Evaluation indicators commonly used in LCA models will be used to evaluate our models, such as the Akaike Information Criterion, Bayesian Information Criterion (BIC), and sample-size-adjusted box BIC (aBIC). It is believed that the accuracy of using BIC for evaluation is higher when the sample size is large; therefore, we have chosen BIC for the evaluation of this index (52). The smaller the value, the better the fitting degree of the model. Classification accuracy will be evaluated using entropy, which ranges from 0 to 1; the closer the value is to 1, the more accurate the classification (54). Finally, the Lo–Mendel–Rubin and bootstrap-based likelihood ratio tests will be used to evaluate the fitting differences of the LCA models. Statistical significance is set at *p* < 0.05.

#### 2.4.4 Interpretation of the results

Once the LCA model is determined, each participant will be assigned to one of the need pattern categories with the highest probability. Through LCA, we will answer our study questions (i) and (ii) and provide a scientific explanation to identify the multiplicity and latent class of rehabilitation needs of older adults in the community and the distribution proportion of each category.

#### 2.4.5 Exploration of influencing factors

Differences in the characteristics of respondents in different categories of demand patterns will be analysed using the Kruskal–Wallis rank tests (for ordinal measurements), one-way analysis of variance (for interval measurements), or chi-square tests (for nominal measurements). We will also perform a polynomial regression analysis to determine the factors that influenced the categories of needs of older adults, primarily by dividing the potential classes of rehabilitation of older adults obtained by LCA into dependent variables. According to the theoretical framework, relevant covariates will be included to analyse the influencing factors of the older adults’ CBR needs. The influencing factors in the theoretical framework will determine the dimensions of the collection of covariates. The covariates and their allocations are listed in Table 3.

**Table 3:** Covariates included in the analysis of influencing factors of older adults’ community-based rehabilitation needs.

Covariate	Definition/Measuring tools	Variable type	Units/categories
**Individual characteristics**			
Gender	Older adult’s gender	Categorical	1 = Male, 2 = Female
Age	Older adult’s age	Continuous	Years (roughly divided into 3–4 categories)
Ethnicity	Older adult’s ethnicity	Categorical	1 = Han ethnicity 2 = Other ethnicity
Education level	Current highest level of education	Categorical	1 = Primary education or below 2 = Junior high school 3 = Senior high school 4 = University or college above
Marital status	Current marital status	Categorical	1 = Married 2 = Divorced 3 = Widowed 4 = Never married
Number of children	Number of living children	Categorical	1 = 1 2 = 2 3 = ≥3
Employment status	Employed status in the past year	Categorical	1 = Formal work 2 = Informal work 3 = Retirement
Living resident	Current residence of older adults	Categorical	1 = City community 2 = Urban–rural fringe community 3 = Countryside community
Income groups	Personal income in the past year	Categorical	1 = Low income 2 = Lower middle income 3 = Middle income 4 = Upper middle income 5 = High income
Source of income	Source of the primary income	Categorical	1 = Pension 2 = Work income 3 = Relatives support 4 = Salary 5 = Other
Medical insurance	Medical insurance types	Categorical	1 = Basic Medical Insurance 2 = Commercial Medical Insurance 3 = No Medical Insurance
Having chronic disease	Suffering from chronic diseases	Categorical	1 = Yes 2 = No
Number of chronic diseases	Number of existing chronic diseases	Categorical	1 = 1 kind 2 = 2 or more
Types of chronic diseases	Classification of chronic diseases	Categorical	1 = Respiratory diseases 2 = Cardiovascular diseases 3 = Cerebrovascular diseases 4 = Digestive system diseases 5 = Metabolic diseases 6 = Other
**Context characteristics**			
Community rehabilitation facilities	The community’s facilities fulfil essential rehabilitation requirements.	Categorical	1 = Satisfy, 2 = Not satisfy
Distance to rehabilitation institution	Distance to the nearest rehabilitation institution	Categorical	1 = Suitable (<2 km) 2 = Relatively far (2–4 km) 3 = Very far (>4 km)
Awareness of rehabilitation services	Knowledge of rehabilitation services provided in the community	Categorical	1 = Yes, 2 = No
Rehabilitation service fee	Evaluation of rehabilitation service fee standard	Categorical	1 = Very low 2 = Relatively low 3 = General 4 = Relatively high 5 = Very high
Barrier-free environment	The environment is designed to be accessible and accommodating to needs	Categorical	1 = Yes, 2 = No
**Health behaviours**			
Demand rehabilitation service	Proactively seek community-based rehabilitation services	Categorical	1 = Yes, 2 = No
Medical treatment	Received any active rehabilitation medical treatment in the past year	Categorical	1 = Yes, 2 = No
**Outcome**			
Health status	Self-assessment of health status	Categorical	1 = Poor 2 = Moderate 3 = Good
Satisfaction	Rehabilitation service satisfaction	Categorical	1 = Very dissatisfied 2 = Not very satisfied 3 = Average 4 = Relatively satisfied 5 = Very satisfied
Self-care ability	ADL scale measurement	Categorical	1 = Complete self-care 2 = Decreased self-care ability 3 = Self-care disability
Frail	TFI scale measurement	Categorical	1 = Yes, 2 = No

## 3 Discussion

Researchers have increasingly focused on intergroup heterogeneity, aiming to enhance the precision and customisation of care and medical treatment by delving into individual differences within groups. While current studies have uncovered variations in the health behaviours of older adults (55) and the heterogeneity of homebound status (56), they have yet to address discrepancies in the needs of older adults in CBR. The need for a greater comprehension of the requirements of older adults’ CBR could significantly impede its effectiveness. With the rapid growth of China’s ageing population and the increasing emphasis on CBR (10), a limited understanding of the needs of older adults in CBR may seriously affect its effectiveness.

To our knowledge, this will be the first study to comprehensively understand the heterogeneity and different need patterns of older adults. Using LCA, we will group older adults with similar needs into the same group. We will identify the rehabilitation needs of older adults with similar community characteristics to better develop targeted interventions and avoid oversimplification and bias in subjective judgement. This will facilitate targeted investments to improve the efficiency and effectiveness of CBR services. Furthermore, by addressing the factors affecting older adults’ needs, individuals can better understand their own needs and seek more effective rehabilitation assistance.

The study will be conducted in Guizhou Province, a region in western China with lower economic levels, fewer medical resources, and less social development than the eastern and coastal areas. The findings of our study will be of great importance to other Chinese provinces with social development similar to that of Guizhou. Furthermore, it can serve as a reference for developing countries facing similar social challenges.

This study has some limitations. First, this study will be conducted in western China, and the results may explain the older adults’ CBR needs in areas with moderate and low development levels. However, there may be differences in older adults’ CBR needs when trying to further extrapolate the results to relatively more developed areas under the influence of a better healthcare system and social development level. At the same time, the data of this study will be collected through questionnaires, which may have subjective biases. However, using a 5-point Likert scale in the questionnaire may reduce some biases.

The Chinese government has completed the construction of CBR services for older adults at the national strategy level (57). We sincerely hope that our study will help older adults receive more accurate CBR services, thus improving their quality of life and health conditions, reducing their burden of chronic disease symptoms, and decreasing their readmission rates and rehabilitation service costs, helping us move toward more inclusive community development (10).

## Ethics statement

The studies involving humans were approved by the Medical Ethics Committee of the Affiliated Hospital of Zunyi Medical University. The studies were conducted in accordance with the local legislation and institutional requirements. Written informed consent for participation in this study was provided by the participants’ legal guardians/next of kin. Written informed consent was obtained from the individual(s) for the publication of any potentially identifiable images or data included in this article.

## Author contributions

LX: Writing – original draft, Conceptualization, Methodology, Data curation. CX: Writing – original draft, Methodology, Visualization. KY: Writing – original draft, Conceptualization. LC: Writing – review & editing. XC: Writing – review & editing. XX: Writing – original draft, Data curation, Methodology. JW: Writing – original draft, Conceptualization, Methodology. XW: Writing – original draft, Methodology. LW: Writing – review & editing, Methodology, Supervision, Funding acquisition.
